# Laparoscopic resection of retroperitoneal paraganglioma close to caudal vena cava in a dog

**DOI:** 10.1002/vms3.588

**Published:** 2021-07-26

**Authors:** Young Tae Park, Tomomi Minamoto

**Affiliations:** ^1^ Ve. C. Jiyugaoka Animal Medical Center Meguro Japan; ^2^ Evergreen Vet Research & Publication Ichinomiya Japan

**Keywords:** caudal vena cava, dog, laparoscopic surgery and paraganglioma

## Abstract

**Objective:**

To report laparoscopic resection of retroperitoneal paraganglioma close to the caudal vena cava in a dog.

**Study design:**

Case report.

**Animal:**

Twelve‐year‐old, neutered male Jack Russell terrier.

**Methods:**

The dog had undergone three previous cystotomies for bladder stones. On follow‐up ultrasonographic evaluation, a 14‐mm × 17‐mm tumour was incidentally detected in the dorsal midline of the caudal abdomen. The dog underwent computed tomography (CT) imaging and ultrasound‐guided fine needle aspiration of the tumour under general anaesthesia. CT imaging showed that the tumour was close to the caudal vena cava. There was no evidence of metastasis. Neuroendocrine tumour was suspected on cytologic examination. Based on these findings, laparoscopic tumour resection was performed using a vessel‐sealing device. The operation time was 136 minutes.

**Results:**

The dog was stable after recovery from anaesthesia and discharged to home the next day. Histopathological diagnosis of the tumour was a paraganglioma. The dog remained without clinical evidence of recurrent tumour or metastasis for 670 days after the surgery.

**Conclusion:**

Retroperitoneal paraganglioma in dogs is uncommon, but it is one of the differential diagnoses of a retroperitoneal tumour. Laparoscopic resection of a retroperitoneal paraganglioma was successfully performed in the dog. Laparoscopic resection conferred the advantages over open surgery of being minimally invasive, providing better visualization of the surgical field through pneumoperitoneum and semisternal patient recumbency, and allowing for magnification of the operative field, which facilitated the ease and safety of the procedure.

## Graphical abstract

Laparoscopic resection of a retroperitoneal paraganglioma close to the caudal vena cava was successfully performed in a dog. The dog remained without clinical evidence of recurrent tumour or metastasis for 670 days after the surgery.

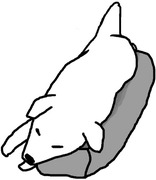

## 1 INTRODUCTION

Paragangliomas are relatively rare in dogs and originate from the paraganglia of the autonomic nervous system (Hines et al., 1993; Rizzo et al., 2008). In dogs, limited information is available regarding paragangliomas, and their classification has not been well established as it has in humans. In humans, paragangliomas originating from the thoracic and abdominal cavity are known to be derived from catecholamine‐producing chromaffin cells, causing various clinical sings, such as tachycardia and hypertension (Eisenhofer et al., 2013). However, human paragangliomas originating from the head and neck are derived from nonchromaffin cells, which do not produce catecholamines (Eisenhofer et al., 2013). Among the tumours derived from catecholamine‐producing chromaffin cells, tumours originating from the adrenal medulla are called pheochromocytomas in humans and dogs (Galac & Korpershoek, 2017; Pacak & Eisenhofer, 2007). In dogs, paragangliomas often originate from the aortic body or carotid body and do not produce catecholamines (Yanagawa et al., 2014). When they arise from the aortic body, they are primarily located at the base of the heart (Capen, 2007). However, the biochemical behaviour of a paraganglioma originating from the abdominal cavity is still unknown in dogs (Mai et al., 2015; Mascort & Pumarola, 1995; Rizzo et al., 2008). In addition to catecholamine production, paragangliomas can cause various clinical signs by compression of the surrounding tissues due to tumour growth, local infiltration and metastasis (Galac & Korpershoek, 2017). In previous reports, dyspnoea and circulatory disorders occurred secondary to the enlargement of the carotid body tumour and compression of the surrounding tissues (Mai et al., 2015; Obradovich et al., 1992). Others reported that local infiltration of paragangliomas into the spinal cord and skull resulted in facial nerve paralysis, dysphagia, Horner's syndrome and quadriplegia. Infiltration close to large vessels, such as carotid vessels, makes surgical resection challenging (Duconseille & Louvet, 2015; Hines et al., 1993; Obradovich et al., 1992; Rizzo et al., 2008). These tumours have been reported to metastasize to the liver, lungs, kidneys, lymph nodes, pancreas, adrenal cortex, brain and bones (Capen, 2007; Cho et al., 1998; Okajima et al., 2007; Yates et al., 1980). The curative treatment option for paraganglioma is surgical resection, and whether the tumour is resectable depends on its location and size (Deim et al., 2007; Rizzo et al., 2008; Wey & Moore, 2012; Yanagawa et al., 2014).

The prognosis of any paraganglioma after surgical resection has not been well documented. A previous study reported that 4 of 10 dogs died within 2 weeks of surgical resection of a carotid body tumour (i.e., paraganglioma), and the median survival time of the remaining six dogs was 25.5 months (Obradovich et al., 1992). Two case reports described the outcome of surgical resection of paraganglioma (Buchanan et al., 1998; Rodrigues et al., 2020). A dog with a left atrium paraganglioma survived about 2 years after surgery. Another dog with paraganglioma of the tongue remained clinically well after surgery for at least 6 months. While rarely found in the caudal mediastinum or retroperitoneum (Rizzo et al., 2008; Robat et al., 2016), a retroperitoneal paraganglioma diagnosed on post‐mortem examination has been previously reported (Dorn et al., 1985; Ilha & Styer, 2013; Robat et al., 2016).

There are no reports regarding the prognosis of surgical resection of a retroperitoneal paraganglioma, including laparoscopic resection, in dogs. The objectives of this case report are to describe the minimally invasive surgical removal of a retroperitoneal primary paraganglioma in close proximity to the caudal vena cava and abdominal aorta in a dog and the long‐term clinical outcomes of the surgery.

## 2 MATERIALS AND METHODS

### 2.1 History

A 12‐year‐old, neutered male, 5.4 kg Jack Russell terrier presented for routine vaccination and a follow‐up ultrasonographic examination. The dog had undergone three cystotomies in the past 5 years for calcium oxalate bladder stones and abdominal ultrasonography had been performed every 4 months to monitor the recurrence of bladder stones. Of note, liver enzymes (i.e., alanine aminotransferase and alkaline phosphatase) were mildly elevated around the time of cystotomy 2 years before, and laparoscopic liver biopsy was performed at the time of cystotomy. Histopathological examination revealed primary hypoplasia of the portal vein. No clinical signs associated with primary hypoplasia of the portal vein had been observed.

### 2.2 Clinical findings and diagnosis

Physical examination findings were unremarkable. Temperature, heart rate, and respiratory rate were 38.2°C, 102 bpm, 20 breaths per minute, respectively. On ultrasonographic examination, a 14‐mm × 17‐mm tumour was incidentally found near caudal vena cava (CVC) and abdominal aorta on the dorsal side of the abdomen.

No other abnormal findings were detected. Based on the ultrasound findings, complete blood count, plasma blood chemistry panel, blood coagulation tests, abdominal ultrasonography, echocardiography, chest and abdominal radiography, and urinalysis were performed prior to anaesthesia. Plasma chemistry panel revealed mild increases in alanine aminotransferase concentration (217 U/L; reference interval [RI], 20–99 U/L) and alkaline phosphatase concentration (712 U/L; RI, 49–298 U/L). Abdominal ultrasonography performed before general anaesthesia showed the retroperitoneal tumour, which was unchanged in size from 12 days earlier, as well as bladder stones. The results of other blood tests, echocardiography, radiography, and urinalysis were unremarkable.

To further define the anatomical location of the tumour and to assess for metastasis, whole body computed tomography (CT) imaging was performed 12 days after the initial examination. Ultrasound‐guided fine needle aspiration (FNA) of the mass were also performed under general anaesthesia immediately after CT imaging.

Prior to anaesthesia, a 22‐gauge peripheral intravenous catheter was placed in the cephalic vein. Anaesthesia for the CT and ultrasound‐guided FNA procedures began with midazolam (0.2 mg/kg) and butorphanol (0.1 mg/kg) administered intravenously as preanesthetic medications. Propofol 6 mg/kg was infused intravenously to effect to induce general anaesthesia, after which the dog was intubated with a 6‐mm diameter endotracheal tube. A surgical plane of anaesthesia was maintained with sevoflurane. CT (Revolution ACT, GE Health Care, Japan) used a 16‐row, 32‐slice device.

The dog was positioned in dorsal recumbency. Noncontrast CT of the head, chest, and abdomen was performed with a slice thickness of 1.25 mm. After the whole body was scanned, a contrast medium (iodine 300 mg/ml) was administered at 2 ml/kg intravenously over 15 s using a power injector (Smart Shot, Nemoto). Immediately after iodine administration, images of the liver to the area around the tumour in the caudal abdomen were obtained in the arterial phase. The portal vein phase and equilibrium phase of the same area were obtained 40 and 120 s after the initiation of iodine administration, respectively. After completion of the CT scan, FNA of the retroperitoneal tumour was performed under ultrasound guidance using a 23‐gauge needle. Cytology slides were prepared and stained with Wright‐Giemsa stain.

**FIGURE 1:**
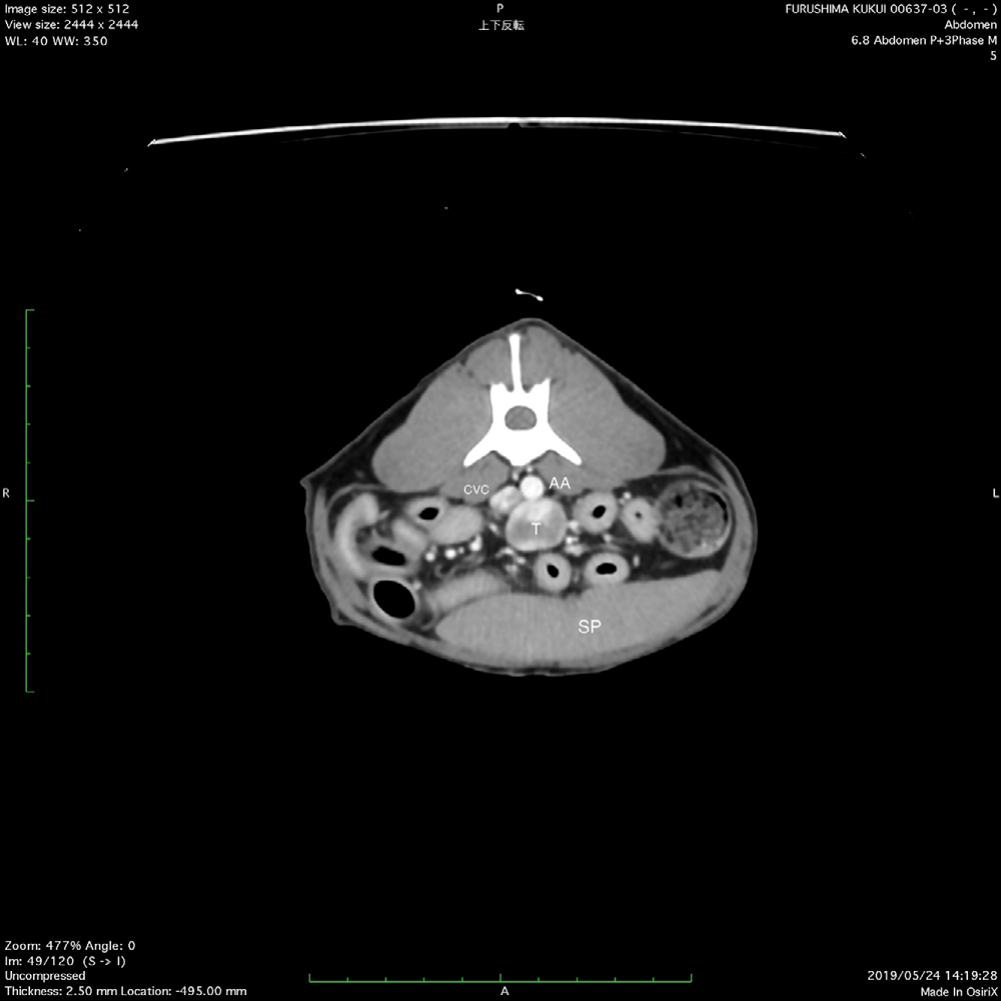
Preoperative computed tomography image. The retroperitoneal tumour was located close to the CVC and AA. AA: abdominal aorta; CVC: caudal vena cava; SP: spleen; T: tumour

Based on CT images, the tumour (14 mm ×17 mm× 19 mm) was located on the midline caudal to the kidney and ventral to the abdominal aorta and caudal vena cava (Figure 1). CT with contrast images showed heterogeneous enhancement of the retroperitoneal mass, with no evidence of vessel invasion. No metastases were noted.

On cytologic examination, many cells with naked nucleation were observed, reflecting cell degeneration. The differential diagnosis included lymphoma, neuroendocrine tumour, or metastatic tumour.

### 2.3 Surgical technique

Three weeks later, laparoscopic tumour resection was performed to completely excise the gross tumour and achieve a histopathologic diagnosis. Prior to aesthetic induction, a 22‐gauge peripheral intravenous catheter was placed in the cephalic vein. Midazolam (0.2 mg/kg) was administered intravenously as a preanesthetic medication and meloxicam (0.2 mg/kg) was administered subcutaneously as an analgesic. Propofol (6 mg/kg) was infused intravenously to effect to induce anaesthesia. The dog was then intubated with a 6‐mm endotracheal tube and connected to a ventilator. Sevoflurane (2.0–3.0%) inhalation was used to maintain a surgical plane of anaesthesia. Intravenous constant rate infusion of fentanyl (5 μg/kg/hr) and ketamine (0.4 mg/kg/hr) were administered as analgesics during surgery. Ampicillin (25 mg/kg) was administered intravenously as a perioperative antibiotic.

**FIGURE 2:**
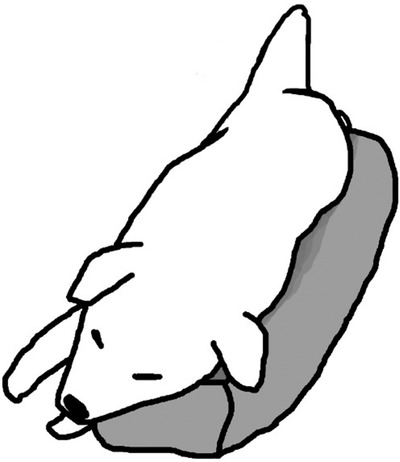
Surgical positioning. The dog was placed in an oblique position intermediate between the lateral position and the prone position by placing cushions and towels under the body

End‐tidal carbon dioxide was maintained at 35–45 mm Hg by adjusting the respiratory rate. The dog was placed in left lateral recumbency with the spine elevated to create a semisternal position by placing cushions and towels under the body so that the dog was tilted toward the side on which the surgeon was standing (Figure 2) (Mayhew & Jolle, 2015).

**FIGURE 3:**
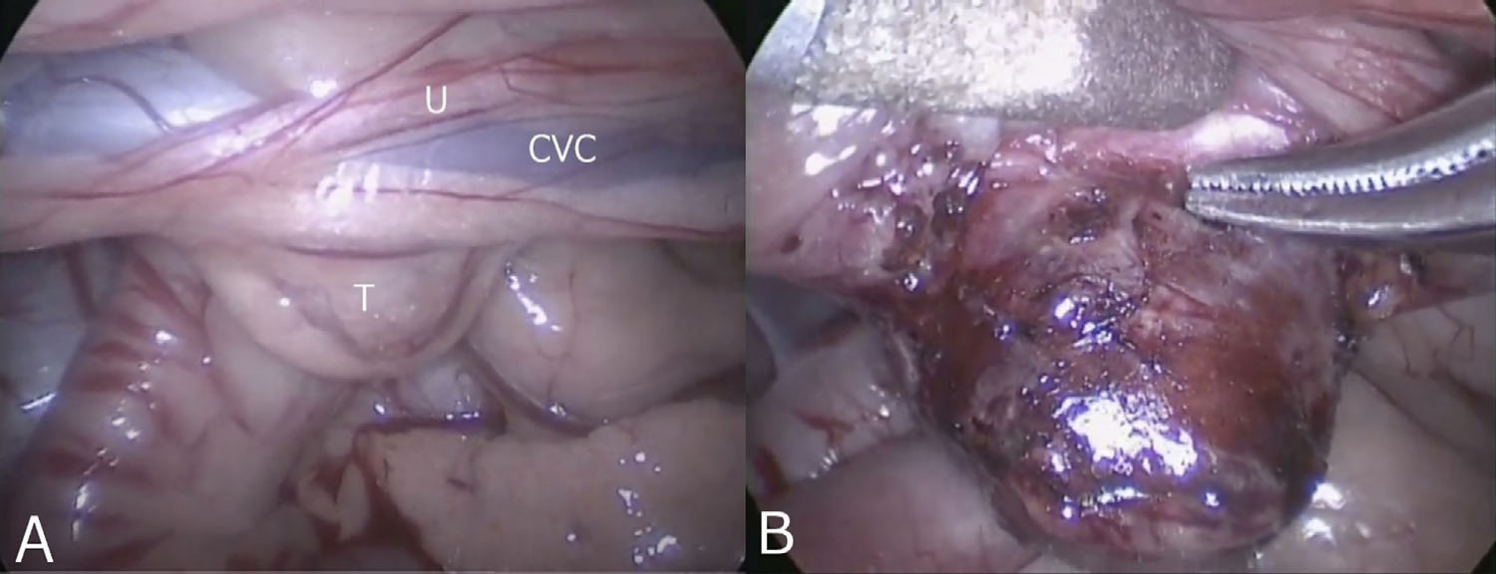
Interoperative laparoscopic images. A. The retroperitoneal tumour was attached to the ventral side of the CVC. B. The tumour was carefully dissected from the large blood vessels while elevating these vessels with a dissector probe. CVC: caudal vena cava; T: tumour; U: right ureter

Based on CT images, a 5‐mm incision in the right flank midway between dorsal and ventral was made as a camera port so that it faced the tumour directly. A 5‐mm cannula (5‐mm EndoTIP Cannula, Karl Storz Endoscopy, Tokyo, Japan) was placed through the camera port using the modified Hasson technique. Pneumoperitoneum was induced by insufflation of CO_2_ (ENDOFLATOR, Karl Storz Endoscopy) at 1.0 L/min, and the intra‐abdominal pressure was maintained at 8 mmHg. A 5‐mm telescope (0° rigid telescope, Karl Storz Endoscopy) was attached to the camera head, and the abdominal cavity was observed through the camera port. After confirming the presence of the tumour, a 5‐mm instrument port was placed both cranial and caudal to the camera port so that the three ports were aligned. The tumour was visualized close to the CVC and abdominal aorta, and the right ureter was identified near the CVC. A small incision was made between the tumour and the CVC using laparoscopic Metzenbaum scissors (CLICKline 5‐mm laparoscopic Metzenbaum scissors, Karl Storz Endoscopy). The tumour was separated from the CVC by blunt dissection using Maryland‐type forceps (5‐mm laparoscopic Maryland type forceps, Karl Storz Endoscopy), a dissector probe (Dissector probe, Hakko co., Ltd, Nagano, Japan), and a vessel sealing device (Sonicision, Covidien Japan, Tokyo, Japan). Large vessels were elevated dorsally with a dissector probe to prevent iatrogenic injury. Bleeding from the tumour was controlled by compression and the vessel‐sealing device (Figure 3). The excised tumour was collected in a specimen retrieval bag (5‐mm Slim Bag Ⅱ, Hakko co., Ltd). To remove the bag from the body, the caudal port was enlarged slightly.

**FIGURE 4:**
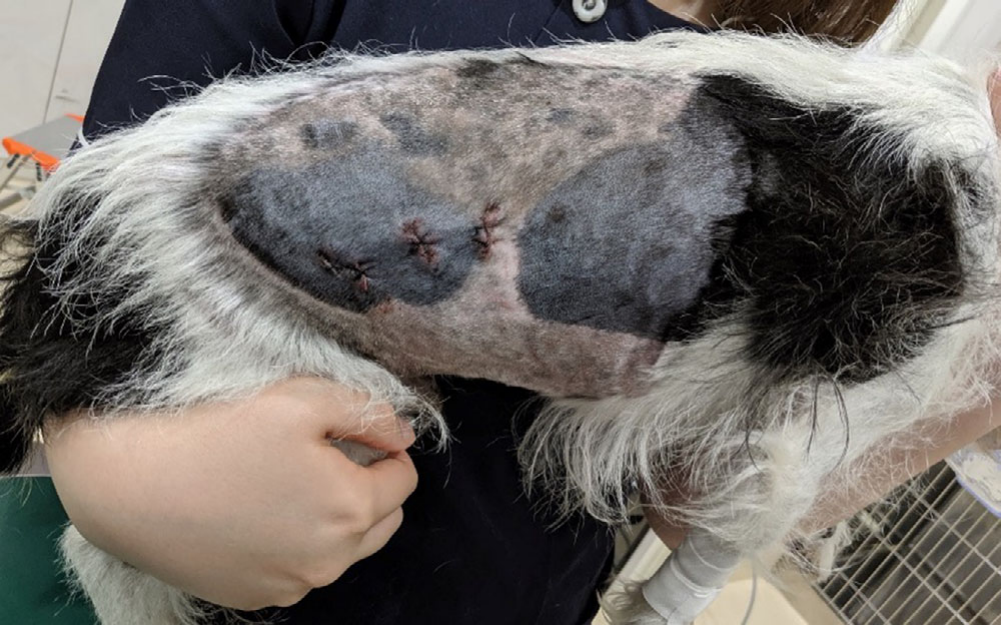
Image of the laparoscopic incision sites immediately after surgery

After verifying the absence of bleeding, the abdominal cavity was de‐sufflated. The abdominal wall and subcutaneous tissue were closed with 3‐0 polydioxanone suture, and the skin was closed with 3‐0 nylon suture (Figure 4). Bupivacaine (1 mg/kg) was injected subcutaneously around the surgical wounds as a postoperative analgesia. The total surgical time was 136 min.

### 2.4 Histopathology

**FIGURE 5:**
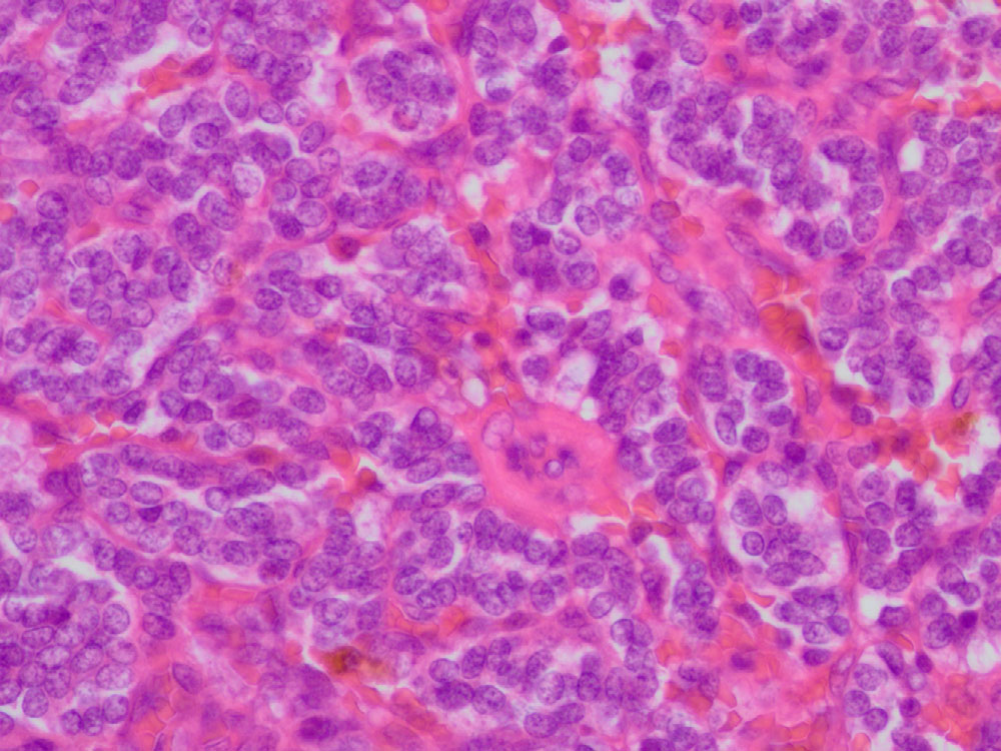
Histopathological image of the excised tumour. Hematoxylin & Eosin stain

**FIGURE 6:**
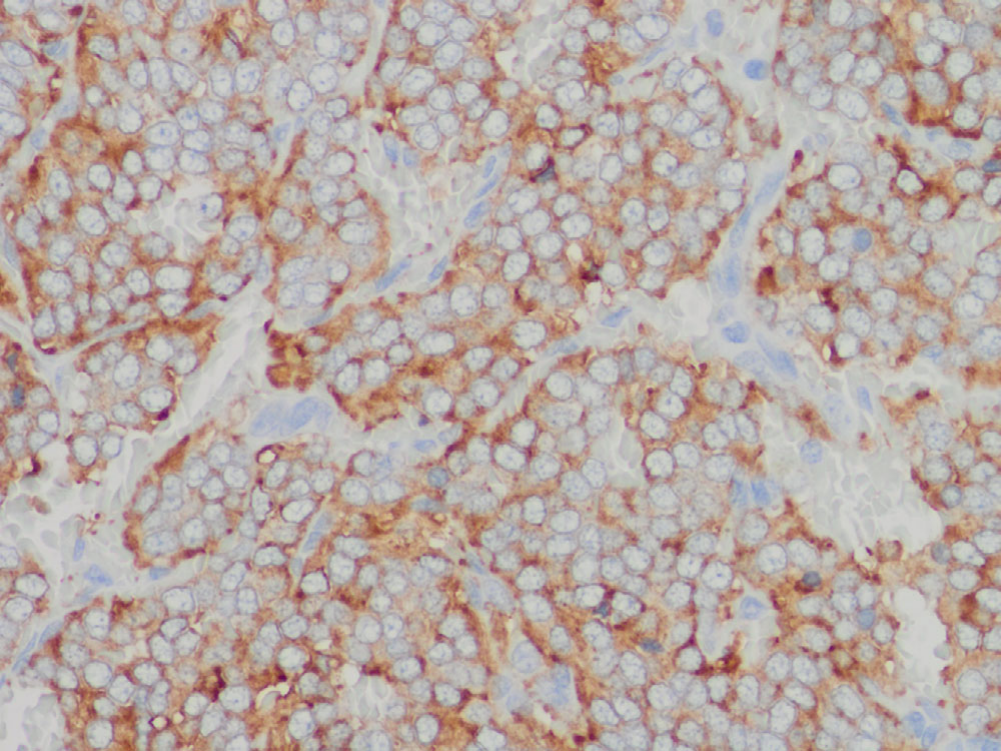
Immunohistochemistry image of the excised tumour. The presence of anti‐chromogranin A antibodies was confirmed

Histopathological examination of the resected tumour revealed alveolar clusters of polygonal cells (Figure 5). On immunohistochemistry staining, the presence of diffuse anti‐chromogranin A antibodies in the plasma was noted, and the tumour was diagnosed as a paraganglioma (Figure 6). Histopathological examination verified complete tumour resection; cell infiltration across the capsule was observed, but there was no obvious intravascular invasion by tumour cells.

## 3 RESULTS

Blood pressure and other vital signs were stable intraoperatively and after surgery. No complications occurred intra‐ or post‐operatively. The patient started eating several hours after the operation, did not seem to care about the surgical wound, and heart rate and respiratory rate were stable. Thus, no additional analgesics were required after surgery, and the dog was discharged home the following day. No post‐operative complications were observed. Follow‐up thoracic and abdominal radiograph and abdominal ultrasonography were performed at 1‐, 3‐, and 6‐month, 1‐year, and 1.8‐year post‐operative periods. No recurrence of the tumour or obvious metastatic findings were noted at the time of last follow‐up 670 days after surgery and the patient remined clinically well.

## 4 DISCUSSION

Retroperitoneal paragangliomas are considered rare as both primary and metastatic tumours and not often associated with biochemical changes, such as catecholamine release in dogs (Dorn et al., 1985; Galac & Korpershoek, 2017; Ilha & Styer, 2013). In this case, no clinical signs were observed, and vital parameters were within normal limits during all physical examinations. In addition, vital signs remained stable during both ultrasound‐guided FNA under general anaesthesia and laparoscopic tumour resection. In humans, arrhythmias, tachycardia, and cardiac arrest secondary to excessive catecholamine secretion have been reported during surgical resection of retroperitoneal paraganglioma (Heinze et al., 2018). It is not fully understood how and when paragangliomas secrete catecholamines in dogs (Dorn et al., 1985; Duconseille & Louvet, 2015; Mascort & Pumarola, 1995).

Surgical resection is the only treatment option for paragangliomas, and complete resection may be achieved depending on the tumour's location and size as well as the presence of metastasis in dogs (Carpenter & Childers, 1988; Wey & Moore, 2012). Ultrasonography is useful for early detection of paragangliomas (Rosenstein, 2000), but in the few previous reports, the tumour had already invaded the surrounding tissues (Barthez et al., 1997), and metastases were observed in multiple organs, including the sentinel lymph nodes, liver, spleen, pancreas, lungs, central nervous system, kidneys and bones (Korpershoek et al., 2019; Okajima et al., 2007; Yanagawa et al., 2014; Yates et al., 1980). As surgical resection should be reserved for dogs without evidence of metastasis, careful preoperative evaluation is necessary to determine whether surgical excision is indicated. Preoperative CT imaging is useful to identify local invasion of tumour and metastasis (Barrera et al., 2013). In the current case, tumour invasion of vessels or surrounding tissues was not observed on either abdominal ultrasonography or CT imaging. There was also no evidence of metastasis to the lymph nodes or lungs. Therefore, surgical resection was indicated.

In humans, laparoscopic resection of retroperitoneal paragangliomas leads to less bleeding and shorter discharge time when compared to conventional open surgery (Ping et al., 2016). In dogs, there have been a few reports of laparoscopic excision of adrenal tumours (Jiménez Peláez et al., 2008; Naan et al., 2013; Pitt et al., 2016). In these reports, laparoscopic adrenalectomy was associated with earlier recovery, shorter hospital stays, fewer wound site complications and shorter surgery time than open surgery. For pheochromocytomas, it has been reported that laparoscopic surgery is suitable for adrenal tumours up to 5 cm in diameter, with no invasion of the CVC (Lang et al., 2011). In the present case, laparoscopic surgery was selected because of its minimally invasive nature and improved visualization of the surgical field over open surgery.

One report described the use of a semi‐sternal position for laparoscopic resection of a right adrenal tumour (Jiménez Peláez et al., 2008). In our current case, we used the same position, which provided a better surgical field by taking advantage of the effects of gravity to shift the surrounding organs away from the surgical site. As a consequence of the pneumoperitoneum and effects of gravity, the intestines around the retroperitoneum did not interfere with the surgical field, and the boundaries between the CVC and the tumour were clear, with the CVC visualized on the dorsal side. The ports were positioned in the right flank to avoid the spleen and stomach, which were located on the left. This surgical approach from the right side provided a good visual field without interference from surrounding organs (Mayhew & Jolle, 2015).

Our laparoscopic approach may be an effective treatment option for resecting retroperitoneal tumours located near large blood vessels. One of the advantages of laparoscopic surgery is the magnified surgical field. Better visualization helps identify blood vessels so that they can be handled with greater caution, which minimizes bleeding from large blood vessels during tumour dissection (Naan et al., 2013).

In previous reports, paragangliomas have caused sudden death in a dog (Obradovich et al., 1992) and were usually diagnosed on post‐mortem examination (Cho et al., 1998; Dorn et al., 1985; Okajima et al., 2007; Yanagawa et al., 2014). In this case, an asymptomatic paraganglioma was found incidentally during follow‐up examination and surgically resected. The patient has been in remission for more than 670 days at the time of this report. Early diagnosis and surgical resection may yield better clinical outcomes in cases without metastasis.

## 5 CONCLUSION

A paraganglioma should be considered as a differential diagnosis of a retroperitoneal tumour in dogs. As surgical treatment is recommended for paragangliomas with no evidence of local invasion or metastasis, laparoscopic resection could be reasonably considered based on a magnified surgical field and its minimally invasive nature. The recommended approach involves semi‐sternal position and insufflation to ensure a good laparoscopic field of view. Further research is necessary to investigate the criteria for defining resectability, the frequencies of functional or non‐functional tumours, how catecholamine secretion affects surgical resection, and the long‐term prognosis after surgery.

## CONFLICTS OF INTEREST

The authors declare no conflicts of interest related to this report.

## AUTHOR CONTRIBUTION

Young Tae Park wrote the original draft and Tomomi Minamoto wrote the review and edited.

### PEER REVIEW

The peer review history for this article is available at https://publons.com/publon/10.1002/vms3.588.

## Supporting information

SUPPORTING INFORMATION

## Data Availability

Data sharing is not applicable to this article as no new data were created or analyzed in this study.
